# Conversion of biomass-derived oligosaccharides into lipids

**DOI:** 10.1186/1754-6834-7-13

**Published:** 2014-01-28

**Authors:** Zhiwei Gong, Qian Wang, Hongwei Shen, Lei Wang, Haibo Xie, Zongbao K Zhao

**Affiliations:** 1 Dalian National Laboratory for Clean Energy and Dalian Institute of Chemical Physics, CAS, 457 Zhongshan Road, Dalian 116023, PR China; 2 University of the Chinese Academy of Sciences, Beijing 100049, PR China

**Keywords:** Biodiesel, *Cryptococcus curvatus*, Microbial lipids, Oleaginous yeast, Oligosaccharides, Simultaneous saccharification and lipid production

## Abstract

**Background:**

Oligocelluloses and oligoxyloses are partially hydrolyzed products from lignocellulosic biomass hydrolysis. Biomass hydrolysates usually contain monosaccharides as well as various amounts of oligosaccharides. To utilize biomass hydrolysates more efficiently, it is important to identify microorganisms capable of converting biomass-derived oligosaccharides into biofuels or biochemicals.

**Results:**

We have demonstrated that the oleaginous yeast *Cryptococcus curvatus* can utilize either oligocelluloses or oligoxyloses as sole carbon sources for microbial lipid production. When oligocelluloses were used, lipid content and lipid coefficient were 35.9% and 0.20 g/g consumed sugar, respectively. When oligoxyloses were used, lipid coefficient was 0.17 g/g consumed sugar. Ion chromatography analysis showed oligocelluloses with a degree of polymerization from 2 to 9 were assimilated. Our data suggested that these oligosaccharides were transported into cells and then hydrolyzed by cytoplasmic enzymes. Further analysis indicated that these enzymes were inducible by oligocelluloses. Lipid production on cellulose by *C. curvatus* using the simultaneous saccharification and lipid production process in the absence of cellobiase achieved essentially identical results to that in the presence of cellobiase, suggesting that oligocelluloses generated *in situ* were utilized with high efficiency. This study has provided inspiring information for oligosaccharides utilization, which should facilitate biorefinery based on lignocellulosic biomass.

**Conclusions:**

*C. curvatus* can directly utilize biomass-derived oligosaccharides. Oligocelluloses are transported into the cells and then hydrolyzed by cytoplasmic enzymes. A simultaneous saccharification and lipid production process can be conducted without oligocelluloses accumulation in the absence of cellobiase by *C. curvatus*, which could reduce the enzyme costs.

## Introduction

Lignocellulosic biomass, such as agricultural residues and forestry wastes, has been widely recognized as a sustainable source for biofuels production [1]. However, cellulose and hemicellulose, two major sugar polymers of lignocelluloses, have to be depolymerized by hydrolysis to enable more efficient microbial utilization. Biomass hydrolysates usually contain monosaccharides as well as various amounts of oligosaccharides [2-5]. Oligocelluloses, water soluble oligomers of β-1,4-linked glucose, are the main incomplete hydrolyzed products of cellulose. Oligoxyloses, water soluble oligomers of β-1,4-linked xylose, are incomplete hydrolyzed products of hemicellulose. These oligomers are produced during biomass pretreatment as well as the hydrolysis process and may be further hydrolyzed to monosaccharides by glycosidases. Cellobiose is the simplest form of oligocellulose. It is a stronger inhibitor of cellulase than glucose, and remarkably slows down the rate of cellulose hydrolysis [6,7]. The addition of β-glucosidase is recommended for the removal of cellobiose inhibition [7-9]. The hydrolysis of cellulose remains a major hurdle for the production of biofuels from lignocellulosic biomass [10].

Oligosaccharides are more challenging substrates than monosaccharides for microorganisms, because assimilation of oligosaccharides may require additional hydrolytic enzymes and transportation systems. However, if those oligomers are left over during microbial transformation, major problems occur, such as reduced product yield and increased water pollution. To enable the consumption of oligosaccharides, microbes should secrete or surface-display glycosidase to enable extracellular hydrolysis [11-13], or harbor a dedicated transport system to take up oligosaccharides for intracellular utilization [14,15]. Microorganisms that can directly assimilate biomass-derived oligosaccharides for the production of biofuels or biochemicals would be much more advantageous [5,16-18].

Oleaginous microorganisms accumulate neutral lipids consisting of long-chain fatty acids, comparable to those of vegetable oils, under nutrient-limited conditions [19]. Microbial lipids have been developed as potential substitutes for high value products, such as cocoa butter and polyunsaturated fatty acids [20,21]. Further, oleaginous microorganisms have been cultivated on lignocellulosic sugars and the microbial lipid products are recognized as promising feedstock for the production of second-generation biodiesel [22-26]. In most cases, however, monosaccharides such as glucose and xylose were used as the carbon sources for cell culture [26,27].

The oleaginous yeast *Cryptococcus curvatus* can produce microbial lipids using a mixture of glucose and xylose as well as cellulosic biomass as feedstocks [28,29]. We have developed the simultaneous saccharification and lipid production (SSLP) process for direct conversion of cellulose into lipids by oleaginous species in the presence of cellulase and β-glucosidase [29]. It is conceivable that the costs of the SSLP process can be further reduced by dropping β-glucosidase if the lipid-producing yeast can assimilate oligosaccharides. Here, for the first time, we have demonstrated that *C. curvatus* can utilize either oligocelluloses or oligoxyloses as the sole carbon source for microbial lipid production. Our data suggested that these oligosaccharides were transported into cells and then hydrolyzed by cytoplasmic enzymes. We found that the SSLP process with *C. curvatus* could be done with comparable cellulose conversion and lipid yield in the absence of cellobiase. This study has provided inspiring information for oligosaccharides utilization, which should facilitate more efficient production of biofuels and biochemicals from lignocellulosic biomass.

## Results and discussion

### Lipid production on oligosaccharides by *C. curvatus*

**Figure 1:**
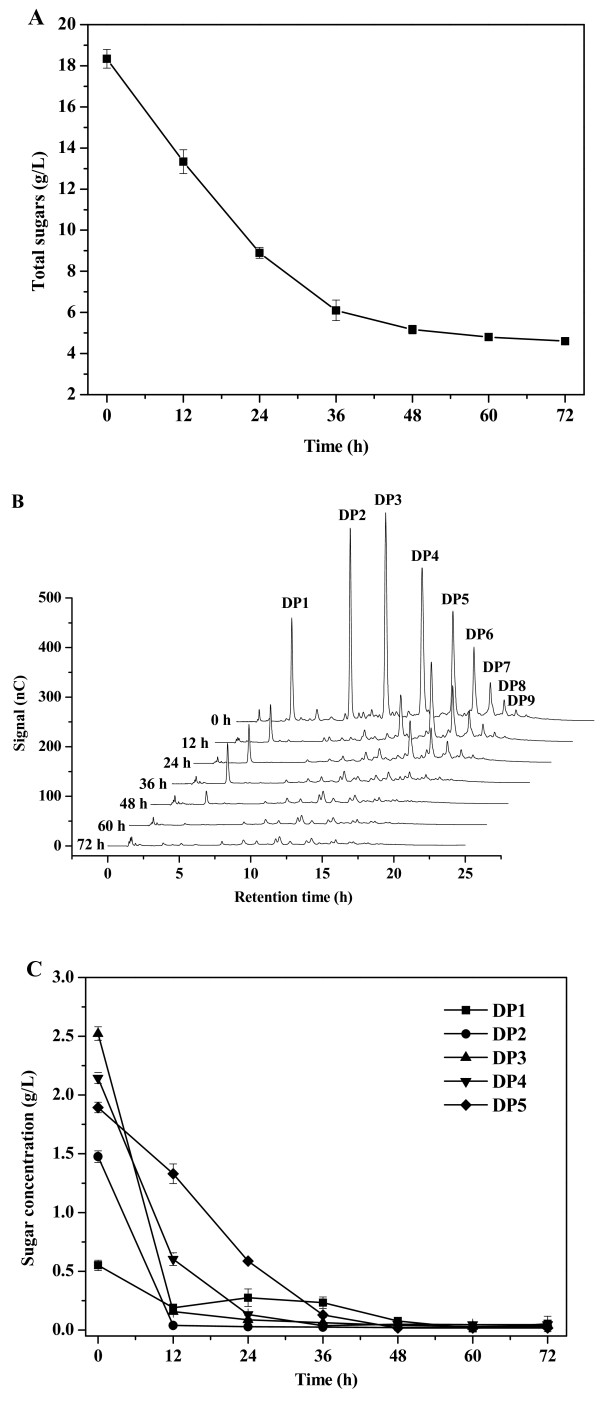
**Time course of oligocellulose consumption by *C. curvatus*. (A)** Total sugar consumption. **(B)** Ion chromatogram of oligocellulose consumption. **(C)** Individual oligocellulose consumption. *C. curvatus* cells were cultured at 30°C, 200 rpm for 72 h, and initial oligocellulose concentration was 20 g total sugar/L. DP1 is glucose. DP, degree of polymerization.

Oligosaccharides are commonly found in biomass hydrolysates yet are difficult to utilize for some microorganisms [3]. We cultivated cells of the oleaginous yeast *C. curvatus* ATCC 20509 using oligocelluloses as carbon sources to identify an advantageous strain for microbial lipid production. Substrate consumption profiles are shown in Figure 1. We found that the total sugar concentration dropped rapidly during the first 36 h before starting to level out (Figure 1A). Ion chromatography (IC) results clearly demonstrated that *C. curvatus* metabolized oligocelluloses with degree of polymerization (DP) 2 to DP9. When the culture was stopped at 72 h, oligocelluloses of DP2 to DP9 were fully consumed (Figure 1B). The results shown in Figure 1C indicate that oligocelluloses with lower DPs were utilized at higher consumption rates, and that these species were consumed simultaneously. After 72 h, residual sugar, cell mass, lipid content and the lipid coefficient were 4.6 g/L, 7.6 g/L, 35.9% and 0.20 g/g consumed sugar, respectively (Table 1). These data suggest that *C. curvatus* realized a comparable lipid coefficient on oligocelluloses to that on glucose. Thus, *C. curvatus* represents a natural yeast strain capable of converting oligocelluloses into lipids with high efficiency.

**Table 1:** **Cultivation results and fatty acid compositions of lipid samples on oligosaccharides by *C. curvatus***

**Carbon source**	**Culture time (h)**	**Residual sugars (g/L)**	**Cell mass (g/L)**	**Lipid yield (g/L)**	**Lipid content (%)**	**Lipid coefficient (g/g consumed sugar)**	**Relative fatty acid content (%)**				
							**Myristic acid**	**Palmitic acid**	**Stearic acid**	**Oleic acid**	**Linoleic acid**
Oligocelluloses	72	4.6 ± 0.1	7.6 ± 0.1	2.7 ± 0.1	35.9 ± 0.3	0.20 ± 0.01	1.1 ± 0.2	45.7 ± 2.3	17.7 ± 1.7	34.2 ± 4.3	-
Oligoxyloses	72	9.1 ± 0.2	5.4 ± 0.0	1.6 ± 0.1	30.3 ± 0.3	0.17 ± 0.01	0.8 ± 0.0	42.1 ± 0.8	13.8 ± 0.3	41.0 ± 1.5	0.9 ± 0.1

**Figure 2:**
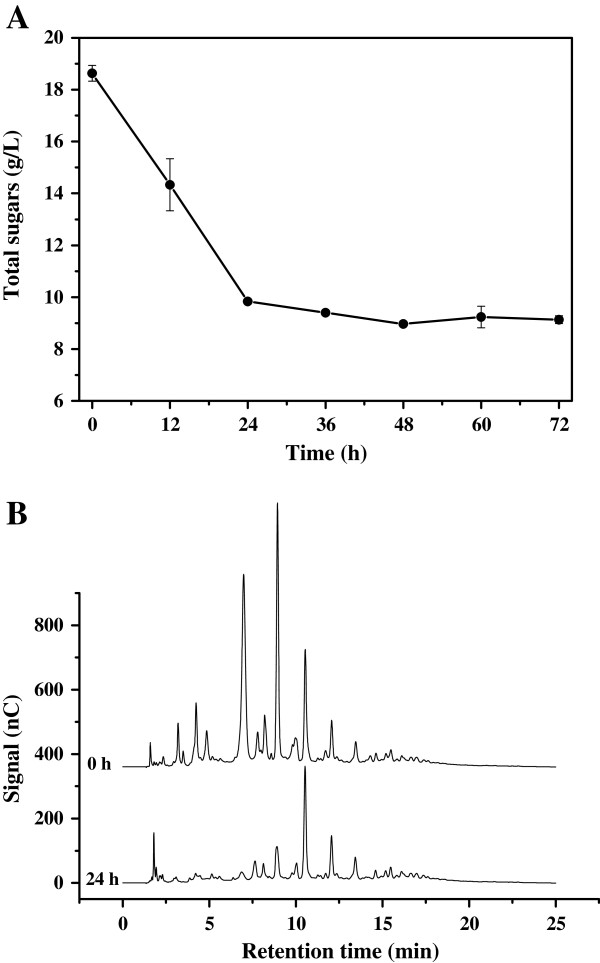
**Time course of oligoxylose consumption by *C. curvatus*. (A)** Total sugars consumption. **(B)** Ion chromatogram of oligoxyloses consumption. *C. curvatus* cells were cultured at 30°C, 200 rpm for 72 h, and initial oligoxyloses concentration was 20 g total sugar/L.

Oligoxyloses were also utilized by *C. curvatus* (Figure 2). About 8.8 g/L of total sugars were consumed during the first 24 h, and almost no further consumption occurred from 24 h to 72 h (Figure 2A). IC results indicated that *C. curvatus* selectively utilized oligosaccharides with lower DPs in the sample. Those with retention time less than 10 min were consumed while others apparently remained intact during the first 24 h (Figure 2B), suggesting that oligoxyloses with higher DPs were difficult substrates for *C. curvatus*. After 72 h, residual sugar, cell mass, lipid content and lipid coefficient were 9.1 g/L, 5.4 g/L, 30.3% and 0.17 g/g consumed sugar, respectively (Table 1). These data suggest that *C. curvatus* is able to realize a comparable lipid coefficient on oligoxyloses to that achieved by the oleaginous yeast *Trichosporon cutaneum* on xylose [30].

The lower lipid coefficient on oligoxyloses suggests that *C. curvatus* probably metabolizes xylose through the pentose phosphate pathway rather than through the phosphoketolase pathway [31]. In an early study, oligoxyloses recovered from stream-exploded wheat straw were consumed by *Microsphaeropsis* sp. for lipid production, since the oleaginous fungus was able to secrete xylanase to degrade oligoxyloses [5]. To use oligoxyloses for ethanol production, *Saccharomyces cerevisiae* strains were engineered expressing β-xylosidase [4,32]. Apparently, *C. curvatus* is exceptional as it can directly utilize both oligocelluloses and oligoxyloses for lipid production. Because biomass hydrolysates usually contain monosaccharides as well as various amounts of oligosaccharides, direct utilization of oligosaccharides for lipid production should further promote full utilization of biomass.

### Localization of oligocellulose-degrading enzymes and aryl-β-glucosidase activity

**Figure 3:**
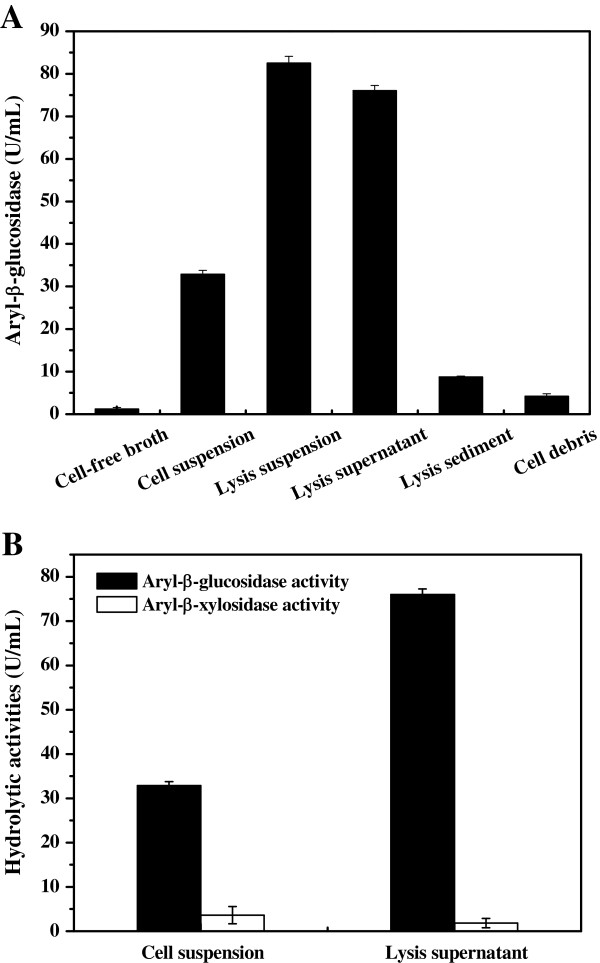
**Hydrolytic activity of different samples. (A)** Aryl-β-glucosidase activity. **(B)** Aryl-β-glucosidase activity and aryl-β-xylosidase activity. *C. curvatus* cells were grown in the oligocelluloses medium for 12 h, and then collected for assay. Aryl-β-glucosidase and aryl-β-xylosidase activities were estimated by using the commercial substrate *p*-nitrophenyl-β-D-glucopyranoside and *p*-nitrophenyl-β-D-xylopyranoside, respectively.

To get a better understanding of the mechanisms of oligosaccharide assimilation by *C. curvatus*, we used *p*-nitrophenyl-β-D-glucopyranoside as a surrogate substrate to quantify aryl-β-glucosidase activities of a variety of sample preparations (Figure 3). Although cells were cultivated using oligocelluloses as the carbon source, aryl-β-glucosidase activity was barely detectable in the cell-free broth sample but reached 32.9 U/mL in cell suspension (Figure 3A). These results suggest that those enzymes are associated with the cells, either located on the cell surface or within the cell. When cells were disrupted and fractionated, aryl-β-glucosidase activities for the lysis suspension, lysis supernatant, cell sediment and cell debris samples were 82.5, 76.0, 8.6 and 4.2 U/mL, respectively. Lysis suspension and lysis supernatant samples showed significantly higher activity than that of the cell suspension sample. However, activities of cell sediment and cell debris samples were an order of magnitude lower than those of lysis suspension and lysis supernatant samples. These data further suggest that aryl-β-glycosidic enzymes are predominantly located in the cytoplasm rather than on the cell wall or bound to cell membranes. The fact that activity within the lysis suspension sample was over 2-fold higher than that of the cell suspension sample implies that oligocellulose transportation might be rate-limiting during oligocelluloses assimilation. When assays were performed using *p*-nitrophenyl-β-D-xylopyranoside as a substrate, significantly lower activities were observed for both cell suspension and lysis supernatant samples (Figure 3B), suggesting that those aryl-β-glycosidic enzymes strongly disfavored the xylopyranoside substrate.

**Figure 4:**
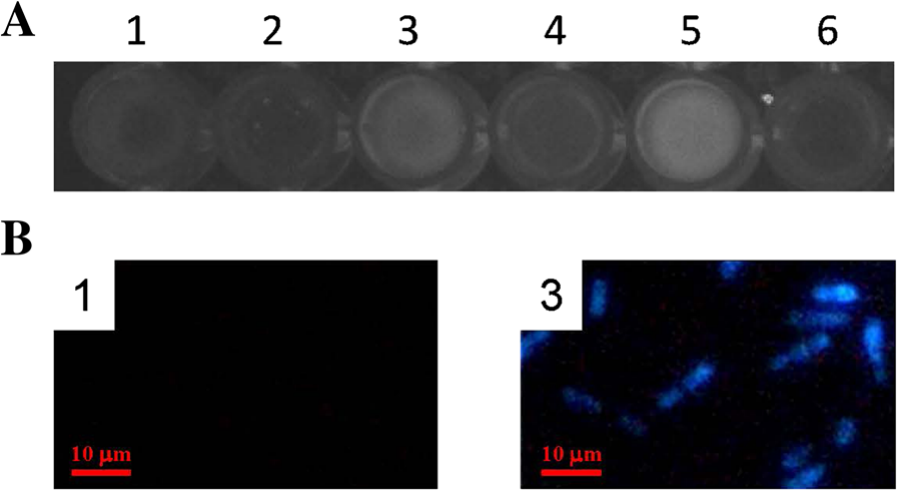
**Microscopy analysis results. (A)** Photograph under UV light at 365 nm. **(B)** Fluorescence microscopy image. A1 and B1, *C. curvatus* cell suspension; A2, MUG solution; A3 and B3, MUG-treated cell suspension; A4, supernatants from A3; A5, wet cells from A3 resuspended in buffer; A6, MUG-treated cell culture supernatants.

We further used 4-methylumbelliferyl-β-D-glucopyranoside (MUG) as a surrogate substrate to investigate the localization of hydrolytic enzymes produced by *C. curvatus* cells. When MUG was hydrolyzed, the product 4-methylumbelliferol was fluorescent. When observed under UV light at 365 nm, cells treated with MUG showed clear fluorescence whereas the supernatants had no such fluorescence (Figure 4A). Further, the treated cells showed up as blue, but cells without MUG treatment were not observed (Figure 4B). These results suggest that MUG was transported into cells and then hydrolyzed by intracellular β-glycosidic enzymes.

### Native PAGE and MUG-zymogram analysis

**Figure 5:**
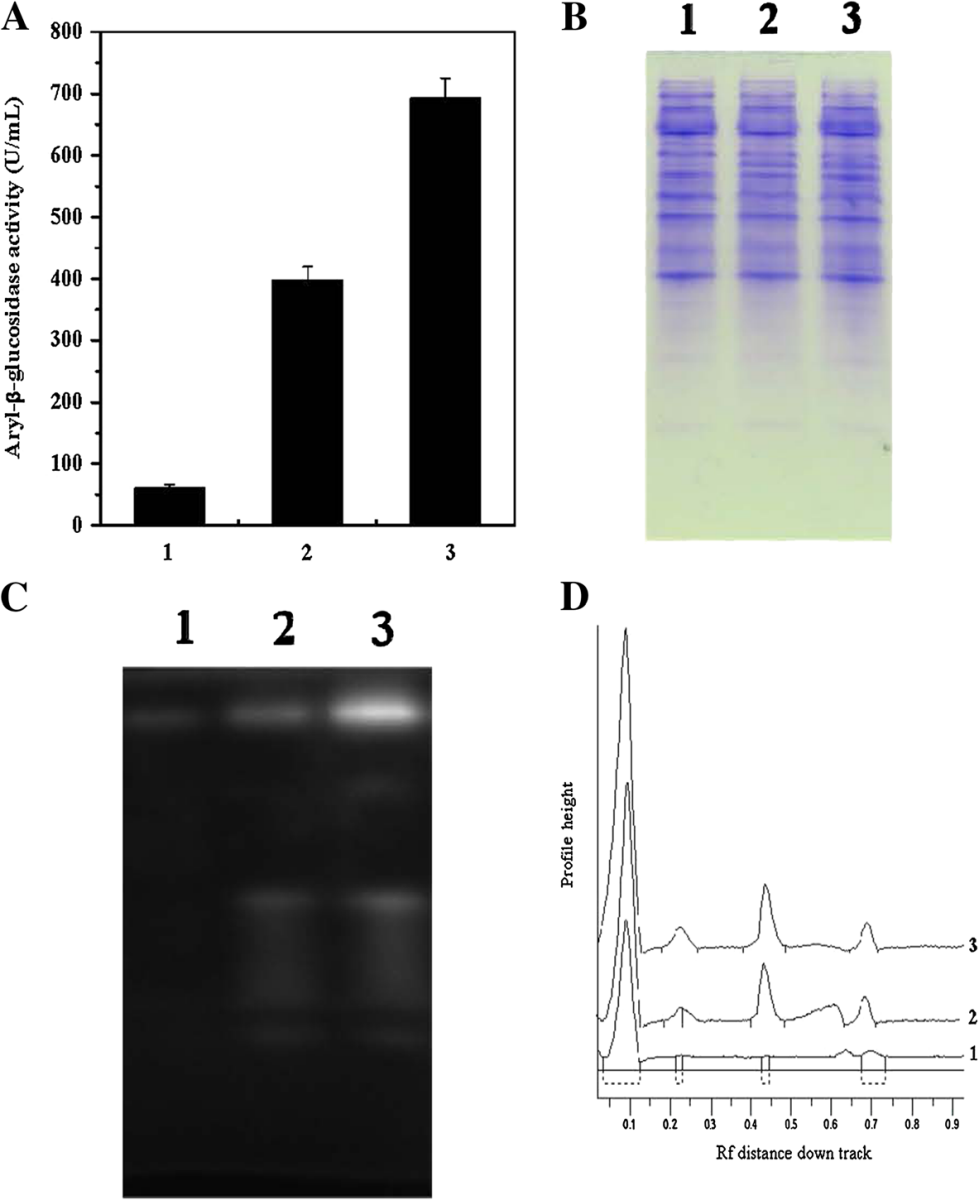
**Analysis of *C. curvatus *cells cultured on glucose (1), cellobiose (2) and oligocelluloses (3). (A)** Aryl-β-glucosidase activity of cell lysis supernatants. **(B)** Native PAGE gel stained with Coomassie brilliant blue R-250. **(C)** Native PAGE gel stained with 5 mM MUG at 37°C for 30 min and visualized under UV light at 365 nm. **(D)** Fluorescence scanning results of the MUG-treated gel.

Because the hydrolytic enzymes were predominantly located in the cytoplasm, the lysis supernatant samples were separated by native PAGE for in-gel β-glucosidase activity detection. *C. curvatus* cells were cultivated for 12 h on glucose, cellobiose and oligocelluloses, respectively, and equal amounts of wet cells were applied for lysis supernatant preparation. Total aryl-β-glucosidase activities of these samples are shown in Figure 5A. When aryl-β-glucosidase activity of the sample prepared from oligocelluloses was set as 100%, activities of samples prepared from cellobiose and glucose were 57.3% and 8.7%, respectively. The fact that aryl-β-glucosidase activities were significantly higher in cells cultured in the presence of oligocelluloses or cellobiose suggested that those glycosidic enzymes were induced by oligocelluloses. Protein staining of the native PAGE gel with Coomassie brilliant blue R-250 found little differences among samples prepared from these three types of carbon sources (Figure 5B). However, when the gel was treated with MUG and then visualized under UV light at 365 nm, at least four bands appeared. The significant intensity differences among these samples (Figure 5C) suggests that different amounts of the fluorescence product, 4-methylumbelliferol, were generated upon hydrolysis of MUG by β-glycosidic enzymes. This was further supported by fluorescence intensity scanning data shown in Figure 5D. Together, our data suggest that β-glycosidic enzymes responsible for oligocelluloses utilization have different isoforms and are inducible by oligocellulose, including cellobiose.

### Conversion of cellulose into microbial lipids by *C. curvatus*

**Figure 6:**
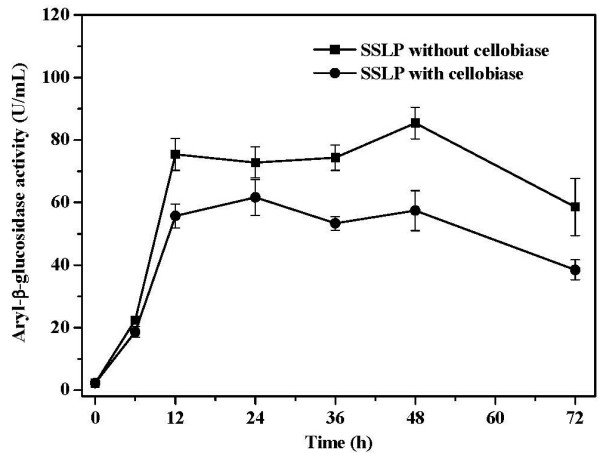
**Time course of cell-bound aryl-β-glucosidase activities during the simultaneous saccharification and lipid production process.** Cells were cultured on cellulose at 30°C, 200 rpm with or without cellobiase. SSLP, simultaneous saccharification and lipid production.

Recently, we showed efficient lipid production on cellulose by *C. curvatus* with the SSLP process [29]. In that study, cellobiase was applied to minimize cellobiose accumulation, because cellobiose is a strong inhibitor of cellulase [6,7,33]. Because we had demonstrated that *C. curvatus* is able to utilize oligosaccharides directly for cell growth and lipid production (*vide ante*), we envisioned that lipid production on cellulose may be achieved without cellobiase loading. When an experiment was done in the presence of cellulase and heat-inactivated cellobiase, the lipid yield and cellulose conversion were 7.0 g/L and 79.3%, respectively, after 48 h. When the cellobiase was active, the lipid yield and cellulose conversion were 7.2 g/L and 87.5%, respectively (Table 2, Entry 1 and 2). These data suggest that the presence of active cellobiase slightly increases initial rates for cellulose hydrolysis and lipid production. However, both lipid yield and cellulose conversion were essentially identical after 72 h regardless of the presence or absence of active cellobiase (Table 2, Entry 3 versus 4). Thus, the presence of additional cellobiase contributed very little to the overall cellulose conversion and lipid yield, although the conversion rate was slower in the absence of active cellobiase. Figure 6 shows the evolution of aryl-β-glucosidase activity of the cell suspension during the SSLP process. Aryl-β-glucosidase activity increased during the early stage, was relatively stable from 12 h to 48 h, and then dropped after 48 h. However, at every time point the sample prepared in the absence of cellobiase had higher aryl-β-glucosidase activity than the sample in the presence of cellobiase, suggesting that the absence of cellobiase promoted the production of hydrolytic enzymes.

**Table 2:** **Cultivation results and fatty acid compositions of lipid samples on cellulose according to the simultaneous saccharification and lipid production process by *C. curvatus***

**Entry**	**Enzyme**	**Enzyme loading (U/g cellulose)**	**Culture time (h)**	**Lipid (g/L)**	**Cellulose conversion rate (%)**	**Relative fatty acid content (%)**					
						**Myristic acid**	**Palmitic acid**	**Palmitoleic acid**	**Stearic acid**	**Oleic acid**	**Linoleic acid**
1^1^	Cellulase	15 FPU	48	7.0 ± 0.1	79.3 ± 1.1	0.3 ± 0.1	28.8 ± 3.6	0.7 ± 0.6	14.2 ± 0.7	54.4 ± 1.9	1.1 ± 0.5
2	Cellulase	15 FPU	48	7.2 ± 0.1	87.5 ± 0.7	0.3 ± 0.0	23.2 ± 2.3	0.2 ± 0.3	10.5 ± 1.0	59.1 ± 1.1	5.6 ± 0.5
	Cellobiase	30 CBU									
3^1^	Cellulase	15 FPU	72	9.0 ± 0.1	95.0 ± 0.3	0.3 ± 0.0	24.6 ± 2.4	-	12.2 ± 1.0	58.9 ± 1.1	3.1 ± 2.0
4	Cellulase	15 FPU	72	8.9 ± 0.1	96.3 ± 0.6	0.3 ± 0.0	24.7 ± 0.4	-	8.9 ± 0.3	59.8 ± 1.2	5.1 ± 0.5
	Cellobiase	30 CBU									

^1^Cellobiase was heat-inactivated in a boiling water bath for 10 min, and was loaded at a ratio of 30 CBU/g cellulose. CBU, cellobiase unit; FPU, filter paper unit.

**Figure 7:**
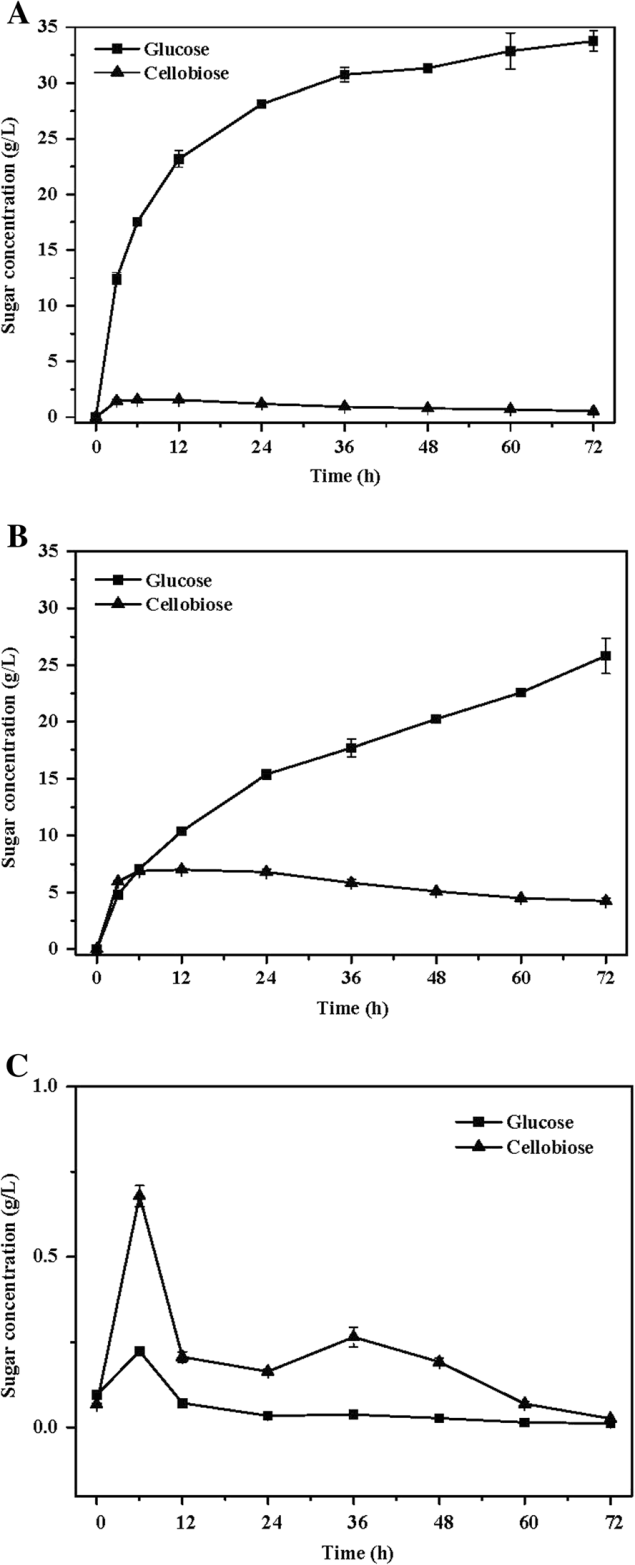
**Time course of glucose and cellobiose evolution during biochemical conversion of cellulose. (A)** Enzymatic hydrolysis by cellulase in the presence of cellobiase. **(B)** Enzymatic hydrolysis by cellulase in the presence of heat-inactivated cellobiase. **(C)** Conversion by *C. curvatus* cells according to the SSLP process with heat-inactivated cellobiase. Initial cellulose concentration was 40 g/L. Cellulase and cellobiase were loaded at 15 FPU and 30 CBU per gram of cellulose. Hydrolysis was done at 50°C, 200 rpm in 0.05 M citrate buffer (pH 4.8). *C. curvatus* cells were cultured at 30°C, 200 rpm. CBU, cellobiase unit; FPU, filter paper unit; SSLP, simultaneous saccharification and lipid production.

To further demonstrate the benefit that *C. curvatus* could offer in biochemical conversion of cellulose, we monitored the evolution of glucose and cellobiose under different conditions (Figure 7). When cellulose was treated with cellulase in the presence of an appropriate amount of *Aspergillus niger* cellobiase, cellobiose was barely accumulated (Figure 7A). However, when cellobiase was heat-inactivated, cellobiose reached 7.0 g/L during the first 12 h, and then gradually dropped to 4.2 g/L at 72 h. Moreover, glucose was produced with a substantial lower rate in the early stage (Figure 7B). These results clearly indicate that the presence of active cellobiase is crucial for efficient hydrolysis of cellulose. Interestingly, cellobiose concentration was always below 0.7 g/L during the SSLP process supplemented with heat-inactivated cellobiase (Figure 7C), suggesting that *C. curvatus* cells produced sufficient hydrolytic enzymes to remove cellobiose. More importantly, there was no oligocellulose accumulation during the SSLP process (data not shown). In sharp contrast, a previous study using a simultaneous saccharification and fermentation process in the presence of cellulase with low cellobiase activity reported that cellobiose reached 10.3 g/L within 72 h when 80 g/L of alkaline-pretreated corn stover was used for ethanol production by *S. cerevisiae*[7]. Thus, in that case, additional cellobiase was used to remove cellobiose and improve ethanol yield. Taken together, *C. curvatus* showed exceptional capacity to metabolize oligosaccharides, which enabled direct yet efficient utilization of cellulose for lipid production in the presence of cellulase only.

### Fatty acid composition profiles

Tables 1 and 2 showed that lipid samples produced by *C. curvatus* contained mainly long chain fatty acids with 16 and 18 carbon atoms. The three major fatty acids were palmitic acid, stearic acid and oleic acid. The fatty acid composition profiles were comparable to those of vegetable oils, suggesting that these products could be explored for biodiesel production [34]. It was interesting to note that lipid samples produced on cellulose by the SSLP process (Table 2) contained substantially more unsaturated fatty acids, especially oleic acid, and less saturated fatty acids, especially palmitic acid, than those obtained using oligosaccharides as substrates without the addition of hydrolytic enzymes (Table 1).

## Conclusions

We have demonstrated for the first time that *C. curvatus* can use oligocelluloses and oligoxyloses directly for lipid production. Our data suggest that these oligosaccharides are transported into the cells and then hydrolyzed by cytoplasmic enzymes. These enzymes were inducible by oligocelluloses. Further, lipid production on cellulose by *C. curvatus* using the SSLP process in the absence of cellobiase achieved essentially identical results to that in the presence of cellobiase. Further work should focus on the elusive oligosaccharide transport system and the hydrolytic enzymes.

## Materials and methods

### Reagents, strain and media

Microcrystalline cellulose with an average particle size of 50 μm was purchase from ACROS (Geel, Belgium). Sigmacell cellulose type 101 was obtained from Sigma and dried to constant weight before use. Oligoxyloses with a DP ranged from 2 to 7 were purchased from Shandong Longlive Biotechnology Co., Ltd (Yucheng, China). Oligocelluloses was prepared from microcrystalline cellulose according to a known procedure [35]. Cellulase from *Trichoderma reesei*, cellobiase from *A. niger* and *p*-nitropehnyl-β-D-glucopyranoside were purchased from Sigma. The activity of cellulase was determined as 161.0 filter paper units (FPU)/mL and 20.3 cellobiase units (CBU)/mL and the activity of cellobiase as 674.7 CBU/mL [36,37]. *p*-Nitrophenyl-β-D-xylopyranoside and MUG were supplied by J & K Scientific Ltd. (Beijing, China). Other reagents used were analytical grade and purchased from a local company.

The oligosaccharide medium contained appropriate amounts of oligosaccharides solution and was supplemented with 0.1 g/L (NH_4_)_2_SO_4_, 1.0 g/L yeast extract, 2.7 g/L KH_2_PO_4_, 2.4 g/L Na_2_HPO_4_ · 12H_2_O, 0.2 g/L MgSO_4_ · 7H_2_O, 0.1 g/L EDTA disodium salt and 1% (v/v) trace element solution, pH 5.5. The composition of the trace element solution contained: 4.0 g/L CaCl_2_ · 2H_2_O, 0.55 g/L FeSO_4_ · 7H_2_O, 0.52 g/L citric acid · H_2_O, 0.10 g/L ZnSO_4_ · 7H_2_O, 0.076 g/L MnSO_4_ · H_2_O and 100 μL of 18 M H_2_SO_4_[38]. The SSLP medium was composed of 40 g/L Sigmacell cellulose type 101, 0.1 g/L (NH_4_)_2_SO_4_, 1.0 g/L yeast extract, 2.7 g/L KH_2_PO_4_, 2.4 g/L Na_2_HPO_4_ · 12H_2_O, 0.2 g/L MgSO_4_ · 7H_2_O, 0.1 g/L EDTA disodium salt and 1% (v/v) trace element solution, pH 5.5. All media were sterilized by autoclaving at 121°C for 18 min before use.

The yeast *C. curvatus* ATCC 20509 was from the American Type Culture Collection center, and maintained at 4°C every two weeks on yeast peptone dextrose agar slants (10 g/L yeast extract, 10 g/L peptone, 20 g/L glucose and 15 g/L agar, pH 6.0). Yeast pre-cultures were prepared from yeast peptone dextrose liquid medium (10 g/L yeast extract, 10 g/L peptone, 20 g/L glucose and pH 6.0) at 30°C, 200 rpm for 24 h.

### Enzymatic hydrolysis of cellulose

The enzymatic hydrolysis of cellulose (Sigmacell cellulose type 101) with 40 g/L in 0.05 M citrate buffer was conducted at pH 4.8, 50°C and 200 rpm. Cellulase and cellobiase were loaded at 15 FPU and 30 CBU, respectively, per gram of cellulose. To check the importance of cellobiase on enzymatic hydrolysis, cellobiase was inactivated in a boiling water bath for 10 min for control experiments.

### Oligosaccharides as a sole carbon source for lipid production

*C. curvatus* cells from 5 mL of pre-cultures were collected by centrifugation, washed twice with 0.85% NaCl, and then inoculated to 50 mL of oligosaccharides medium in 250 mL unbaffled conical flasks. The cultures were held at 30°C, 200 rpm for 72 h.

### Cellulose as a sole carbon source for lipid production

The SSLP process was used to convert cellulose into lipid by *C. curvatus*. Briefly, cells from 5 mL of pre-cultures were collected by centrifugation, washed twice with 0.85% NaCl, and then inoculated to 50 mL of cellulose suspension supplemented with cellulase (15 FPU/g cellulose) and activated or inactivated cellobiase (30 CBU/g cellulose). The culture was held at 30°C, 200 rpm in 250 mL conical flasks for 72 h.

All experiments were done in triplicate.

### Microscope analysis of MUG hydrolysis

*C. curvatus* cells were grown in the oligocellulose medium for 12 h, harvested by centrifugation, washed twice with phosphate citrate buffer, and resuspended in equivalent phosphate citrate buffer before use. Cell samples were incubated with MUG at 37°C for 15 min. Microscopic photographs were acquired by using a color charge-coupled device camera (Nikon, Tokyo, Japan). The treated cells were placed on a glass slide and visualized upon excitation at 330 to 380 nm by an Eclipse 80i fluorescence microscope (Nikon).

### Native PAGE and MUG-zymogram analysis

Native PAGE and MUG-zymogram analysis were carried out according to known methods [39,40] with some modifications. For in-gel β-glucosidase activity detection, crude cell lysate supernatants were analyzed by native PAGE using 10% and 5% polyacrylamide as separation and stacking gels, respectively. Tris-glycine buffer, pH 8.3, was used as the electrode buffer. Electrophoresis was run at a constant current of 10 mA at 4°C for 3 h. Gels were washed with water and 0.2 M phosphate-0.1 M citrate buffer (pH 6.0) before being overlaid with 5 mM MUG in the same buffer, and incubated at 37°C for 30 min. The presence of a fluorescent product was visualized under UV 365 nm and a photograph was acquired using an imaging system (Syngene, UK). Band intensities were quantified by fluorescence scanning (Gene Tools software). Gels were stained with Coomassie brilliant blue R-250 after being photographed under UV light.

### Analytical methods

Aryl-β-glucosidase activity was measured as follows. The reaction mixture was composed of 0.6 mL of phosphate citrate buffer, 0.2 mL of 10 mM *p*-nitrophenyl-β-D-glucopyranoside and 0.2 mL of sample. After incubation at 37°C for 20 min, 2 mL of 1 M Na_2_CO_3_ was added to stop the reaction. The *p*-nitrophenol released was measured spectrophotometrically at 410 nm. One unit of enzyme activity was defined as the amount of enzyme that generated one micromole of *p*-nitrophenol per minute.

Varieties of samples were prepared for aryl-β-glucosidase activity assay. Briefly, *C. curvatus* cells were grown in the oligocellulose media for 12 h, harvested by centrifugation at 6000 × g, 4°C for 5 min, washed twice with the phosphate-citrate buffer (pH 6.0), and resuspended in the same buffer. Both the cell-free broth and cell suspension samples were assayed.

To establish the relationship between catalytic activity and enzyme localization, cells were resuspended in phosphate citrate buffer containing 20 mM EDTA, 1 mM dithiothreitol, 10 mM MgCl_2_ and 50 μg/mL phenylmethylsulfonyl fluoride, ruptured with glass beads by using the FastPrep®-24 homogenizer (MP Biomedicals, LLC., Santa Ana, CA, USA) for 14 cycles of treatment at 6.0 m/s for 40 s and ice-cold for 1 min between intervals. The lysis suspension was centrifuged at 40,000 × g, at 4°C for 30 min to separate the lysis supernatant and cell sediments [41]. The sediments were washed twice before being resuspended and centrifuged at 100 × g, at 4°C for 5 min to separate the intact cells and cell debris. The lysis suspension, lysis supernatant, cell sediments and cell debris samples were used for aryl-β-glucosidase activity assay.

Total sugars were quantified by using the phenol-sulfuric acid method [42]. Oligocellulose mixtures were analyzed by IC on the Dionex ICS2500 system with a CarboPac PA100 guard column (4 mm × 50 mm), a CarboPac PA100 analytical column (4 mm × 250 mm) and an ED50A integrated amperometry detector (Dionex, Sunnyvale, CA, USA). Samples were eluted with the mixture solution of NaOH and NaOAc with gradient elution at a rate of 1 mL/min at 30°C. The injection volume was 25 μL. Sugars were indentified and quantified relative to the standard carbohydrates [43].

Cellulose concentration was determined as described [44]. Residual cellulose in samples was collected by repeated precipitation and washing with water to remove soluble carbohydrates. The precipitated sample was further washed using acetic acid-nitric acid reagent and water to remove non-cellulosic materials [45] and quantified by using the phenol-sulfuric acid method with glucose as the standard [42]. Cellulose conversion was calculated based on the consumed cellulose.

Cell mass, lipid yield, lipid content and lipid coefficient were determined as described [46]. The cell mass was harvested by centrifugation and washed twice with distilled water and determined gravimetrically after drying the wet cells at 105°C for 24 h. The dried cell mass was digested with 4 M HCl at 78°C for 1 h before extraction with chloroform/methanol (1:1, v/v). The extracts were washed with 0.1% NaCl, dried over anhydrous Na_2_SO_4_ and evaporated in a vacuum. The residue was dried at 105°C for 24 h to give the total lipid. Lipid content was expressed as grams of lipid per gram of cell mass. The lipid coefficient was defined as grams of lipid produced per gram of substrate consumed. The fatty acid composition profiles of lipid samples were determined using a 7890 F gas chromatography instrument after transmethylation according to a published procedure [29].

## Abbreviations

CBU: cellobiase unit; DP: degree of polymerization; EDTA: ethylenediaminetetraacetic acid; FPU: filter paper unit; IC: ion chromatography; MUG: 4-methylumbelliferyl-β-D-glucopyranoside; PAGE: polyacrylamide gel electrophoresis; SSLP: simultaneous saccharification and lipid production.

## Competing interests

The authors declare that they have no competing interests.

## Authors’ contributions

ZG designed the study, performed the experiments, analyzed the results and drafted the manuscript. QW participated in the sugar analysis. HS and LW participated in the design of the study and commented on the manuscript. HX commented on the manuscript. ZKZ coordinated the study and revised the manuscript. All authors read and approved the final manuscript.
